# Comprehensive transcriptome analysis of *Crocus sativus* for discovery and expression of genes involved in apocarotenoid biosynthesis

**DOI:** 10.1186/s12864-015-1894-5

**Published:** 2015-09-15

**Authors:** Shoib Ahmad Baba, Tabasum Mohiuddin, Swaraj Basu, Mohit Kumar Swarnkar, Aubid Hussain Malik, Zahoor Ahmed Wani, Nazia Abbas, Anil Kumar Singh, Nasheeman Ashraf

**Affiliations:** Plant Biotechnology Division, CSIR- Indian Institute of Integrative Medicine, Sanat Nagar, Srinagar, J&K-190005 India; Academy of Scientific and Innovative Research (AcSIR), Anusandhan Bhawan, New Delhi, 110 001 India; Stazione Zoologica Anton Dohrn di Napoli, Naples, Italy; Division of Biotechnology, CSIR-Institute of Himalayan Bioresource Technology, Palampur, 176061 India

**Keywords:** *Crocus*, Saffron, Apocarotenoids, Illumina, *De novo* assembly

## Abstract

**Background:**

*Crocus sativus* stigmas form rich source of apocarotenoids like crocin, picrocrocin and saffranal which besides imparting color, flavour and aroma to saffron spice also have tremendous pharmacological properties. Inspite of their importance, the biosynthetic pathway of *Crocus* apocarotenoids is not fully elucidated. Moreover, the mechanism of their stigma specific accumulation remains unknown. Therefore, deep transcriptome sequencing of *Crocus* stigma and rest of the flower tissue was done to identify the genes and transcriptional regulators involved in the biosynthesis of these compounds.

**Results:**

Transcriptome of stigma and rest of the flower tissue was sequenced using Illumina Genome Analyzer IIx platform which generated 64,604,402 flower and 51,350,714 stigma reads. Sequences were assembled *de novo* using trinity resulting in 64,438 transcripts which were classified into 32,204 unigenes comprising of 9853 clusters and 22,351 singletons. A comprehensive functional annotation and gene ontology (GO) analysis was carried out. 58.5 % of the transcripts showed similarity to sequences present in public databases while rest could be specific to *Crocus.* 5789 transcripts showed similarity to transcription factors representing 76 families out of which Myb family was most abundant. Many genes involved in carotenoid/apocarotenoid pathway were identified for the first time in this study which includes zeta-carotene isomerase and desaturase, carotenoid isomerase and lycopene epsilon-cyclase. GO analysis showed that the predominant classes in biological process category include metabolic process followed by cellular process and primary metabolic process. KEGG mapping analysis indicated that pathways involved in ribosome, carbon and starch and sucrose metabolism were highly represented. Differential expression analysis indicated that key carotenoid/apocarotenoid pathway genes including phytoene synthase, phytoene desaturase and carotenoid cleavage dioxygenase 2 are enriched in stigma thereby providing molecular proof for stigma to be the site of apocarotenoid biosynthesis.

**Conclusions:**

This data would provide a rich source for understanding the carotenoid/apocarotenoid metabolism in *Crocus*. The database would also help in investigating many questions related to saffron biology including flower development.

**Electronic supplementary material:**

The online version of this article (doi:10.1186/s12864-015-1894-5) contains supplementary material, which is available to authorized users.

## Background

*Crocus sativus* is a triploid, sterile geophyte which has been cultivated and used as spice and medicinal plant since thousands of years [1]. It is vegetatively propagated by corms which maintains genetic characteristics of the plant but limits its improvement. *Crocus* belongs to family Iridaceae, members of which have relatively large but poorly characterized genomes [2]. *Crocus* genus consists of about 85 species and many of them are considered as economically valuable. The main *Crocus* producing countries are Iran, Greece, Spain, Italy and India (Kashmir). *C. sativus* has 2n = 3X = 24 chromosomes. It is thought to be sterile triploid form of *C. Cartwrightianus* [3]. The desiccated stigma of *C. sativus* forms saffron and is source of various carotenoids and unique compounds called apocarotenoids which are produced by oxidative tailoring of carotenoids [4, 5]. In fact *Crocus* is the only plant which produces apocarotenoids like crocin, picrocrocin and safranal in significant amounts. These compounds provide color, flavour and aroma to this crop making it world’s costliest spice [6]. In addition, these compounds have a broad spectrum of pharmacological properties as well [7, 8].

Since *Crocus* produces important carotenoids and apocarotenoids, it becomes imperative to have a holistic approach for identification and isolation of genes involved in their metabolic pathways. Moreover, carotenoid biosynthesis and degradation is thought to be tightly regulated throughout the life cycle of a plant and dynamic changes appear to occur in their composition so as to stay in tune with prevailing developmental requirements and environmental conditions [9]. Also their regulation is relatively a complex phenomenon [10] with cross talk and integration of various pathways at multiple levels so as to achieve metabolic flexibility and robustness in response to environmental signals. Therefore, transcriptome sequencing would pave way for elucidation of metabolic pathway in *Crocus* and corresponding regulatory networks. Further, the genome organization of *Iridaceae* family is not yet known and therefore transcriptome sequencing of *Crocus* would be first step to provide a gene atlas for other members of this familyas well. The characterization of saffron transcriptome is in fact a prerequisite to shed light on essential biological processes including the molecular basis of flavour and color biogenesis, genomic organization and flower development of *C. sativus* in particular and family *Iridaceae* in general.

Although, a few studies have been carried out on the *C. sativus* plant to understand the flower development and apocarotenoid biosynthesis [2, 11, 12], molecular basis of these essential processes is still largely unknown. This study presents first report on transcriptome sequencing and analysis of *Crocus* stigma and rest of the flower (Flower minus stigma) using high-throughput Illumina sequencing. The assembled transcripts were annotated to identify genes involved in various steps of the carotenoid/apocarotenoid pathway. The transcriptome aids in significant addition to the number of genes which are potentially involved in apocarotenoid biosynthesis. The transcriptome was further screened for the identification of the genes encoding transcription factors (TFs) so as to generate transcription factor database from *Crocus* which may help in unravelling the regulatory mechanism of apocarotenoid biosynthesis. Finally expression specificity of the assembled transcripts provided an accurate estimation of the biological processes involved in different tissues (Stigma or Flower). The *Crocus* transcriptome provides a platform for understanding the molecular basis of carotenoid/apocarotenoid pathway and various other biological processes pertaining to this crop.

## Methods

### Tissue sampling, cDNA library construction and sequencing

*Crocus sativus* L. tissue used in this study was collected from experimental farm at Indian Institute of Integrative Medicine, Srinagar, Jammu and Kashmir, India (longitude: 34°5′24″N; latitude: 74°47′24″ and altitude 1585 m above sea level). The voucher specimen was deposited at Janaki Ammal Herbarium (RRLH), IIIM, Jammu. The details of the specimen are: (Accession number: 22893; Accession date: 12/01/2015; name of collector: Nasheeman Ashraf; Place of collection: IIIM, Srinagar Farm; Date of collection: 01/01/2015). The most important C*rocus* apocarotenoids which include crocin, picrocrocin and saffranal are synthesized and accumulated in stigmas and their amount increases from yellow, through orange to scarlet stage. Also the compounds increase in quantity from pre- anthesis stage to anthesis and then follow a decline post anthesis. In view of this, we collected flowers at scarlet stage. Further, three flowers each were collected at pre-anthesis, anthesis and one day after anthesis. Stigmas were hand-picked from flowers. The tissue samples were frozen in liquid nitrogen and stored at -80 °C for further use. Total RNA was extracted from stigma and rest of the flower using TRIzol (Invitrogen). The quality and quantity of total RNA was analysed using nanodrop spectrophotometer and their integrity was further evaluated using bioanalyzer (Agilent technologies, USA). High quality RNA isolated from three independent tissue samples (biological replicates) was pooled for library preparation. The cDNA libraries of stigma and rest of the flower tissue were constructed using illumina TruSeq RNA preparation kit v2 (Illumina Inc., USA) following manufacturer’s instructions. The libraries were quantified using Qubit dsDNA BR assay kit (Life technologies, USA). The insert size of libraries was verified using bioanalyzer DNA 1000 chip. Further for generating clusters, 10pM of each library was loaded onto the flow cell using TruSeq PE Cluster Kit v5 on cluster station (Illumina Inc., USA). The flow cell containing clonally amplified clusters was loaded onto the Genome Analyzer IIx (Illumina) and paired-end (PE) (2 × 72) sequencing was performed.

The sequence reads which are obtained from sequencer often contain adapter sequences, low quality reads and very short length reads. This data is therefore processed in order to remove such reads. Paired reads were quality filtered using NGS QC toolkit v 2.3 [13]. The cutoff for quality score is > 20 Q30 score and should have high quality bases > 70 % of read length. High quality reads were used for de novo assembly using Trinity software with K-mer of 25. The assembly resulted in contigs and singletons which together form set of unigenes.

### Annotation of the assembled transcriptome

*Crocus* transcriptome was annotated using BLASTx similarity search against NCBI-nr database. Homology search was also made against other databases like Swiss-Prot and Uniprot TrEmBL databases. Further GO term and Interpro domain annotation for the assembled transcripts was performed using the Trapid annotation server (Plaza 2.5 database) [14]. KEGG orthology assignments for the transcripts were obtained using the KAAS server (SBH algorithm) [15]. For identification of transcription factors, homology search was done against PlantTFDB [16]. In all cases analysis was performed using the default parameters.

### Differential expression analysis

The assembled transcripts were filtered to omit sequences < 200 nucleotides. Further Trimmed Mean of M values (TMM) normalization was performed on the raw reads for each transcript using the NOISeq package. Then NOISeq count filter was used to remove transcripts with Counts per Million (CPM) < 1 in both samples which avoids noise from lowly expressed transcripts. Further, the filtered transcripts were analysed with the bioconductor NOISeq package [17] (qvalue cut-off 0.95) to identify those differentially expressed between flower and stigma. The gene ontology enrichment analysis was performed on the GO mapping done by Trapid server step using a custom R script to select significantly enriched GO classes in the differentially expressed transcripts compared to the total transcriptome (minimum representatives for a GO class: 5 transcripts; FDR ≤ 0.05). Finally custom Perl and R scripts were used to associate differentially expressed transcripts with their KEGG identifiers and convert the expression information into a format suitable to be visualized in iPath2 [18].

### Quantitative real time PCR

For real time PCR, total RNA was extracted using TRIzol reagent and used for cDNA synthesis by reverse transcription kit (Fermentas) following manufacturer’s instructions. qRT-PCR was performed in triplicates in ABI StepOne Real time (Applied Biosystems). The reaction was carried out in a total volume of 20 μl which consisted of 10 μL of 2X SYBR Green Master Mix, 0.2 μM gene specific forward and reverse primers and 100 ng of template cDNA. The cycling parameters were 95 °C for 20 s, followed by 40 cycles of 95 °C for 15 s and 60 °C for 1 min. The sequence of the primers used in this study is given in Additional file 1. The relative quantification method (ΔΔ-CT) was used to evaluate quantitative variation between the replicates examined. The amplification of 18S rRNA gene was used as an endogenous control to normalize the data.

### Sequence analysis and phylogenetics

The deduced amino acid sequences of the CCD proteins were aligned by a multiple sequence comparison using the log-expectation (MUSCLE) alignment tool (http://www.ebi.ac.uk/Tools/msa/muscle) with the default parameters [19]. The phylogenetic analysis was done using the neighbor-joining method and 1000 bootstrap replicates were employed in each analysis to maximize the statistical significance [20]. The phylogenetic trees were constructed and visualized by MEGA6.05 software [21]. The accession numbers of the genes used for phylogenetic analysis are given in Additional file 2. All the phylogenetic data including the sequence files, alignment and phylogenetic tree have been submitted to Dryad (doi:10.5061/dryad.k3m55) (http://datadryad.org/).

## Results and discussion

### *Crocus* transcriptome sequencing and *de novo* assembly

The sequencing of *Crocus* cDNA libraries generated 75,432,904 raw reads from flower and 59,043,670 from stigma. Approximately 14 % of raw reads were removed post filtering of adapter sequences, low quality and short reads. Further, 64,604,402 flower and 51,350,714 stigma reads were assembled *de novo* using trinity which resulted in 64,438 transcripts. These transcripts were classified into 32,204 unigenes comprised of 9853 clusters and 22,351 singletons. The statistical summary of data is outlined in Table 1. The average contig length was 609.57 bp, GC content 43.99 % and N50 was 753 bp.

**Table 1:** Summary of *Crocus sativus* transcriptome

Tissue used	Flower	Stigma
No. of raw reads	75,432,904	59,043,670
No. of filtered reads	64,604,402	51,350,714
Total trinity transcripts	64,438	
Contigs	9853	
Singletons	22,351	
Total components	32,204	
Average contig length	609.57	
Contig N50	753	
Percent GC	43.99	

### Functional annotation and classification of *Crocus* transcriptome

For comprehensive annotation of *Crocus* transcriptome, similarity search for the sequences was done using BLASTX against nr database in NCBI, with E- value cutoff of 10^−5^. Interpro, swissprot and Uniprot TrEmBL analysis were also used for annotation. Among the 64,438 transcripts 58.5 % could be annotated. The putative functions assigned to *Crocus* transcripts are available as Additional file 3. *Crocus* belongs to family *Iridaceae* and whole genome and/or transcriptome of none of the members of this family has been sequenced so far and that may be one of the reasons for relatively low homology results. Although EST database of *Crocus* was developed earlier but that reports only 1893 unique transcripts [2]. The sequences with unknown homology may represent genes involved in metabolic processes which are unique to this plant and whose intermediates and enzymes have not been identified so far. Moreover, many transcriptome studies of other plant species have also reported functional annotation of around half or even less percent of unigenes. For example, in case of transcriptome analysis of *Cymbidium sinence*, only 49.88 % of the unigenes could be annotated [22]. Further, significant match could be assigned to only 55 % of the unigenes in bamboo transcriptome [23] while in case of *Amaranthus tricolor* only 52.89 % of the unigenes showed significant similarity [24].

**Fig. 1:**
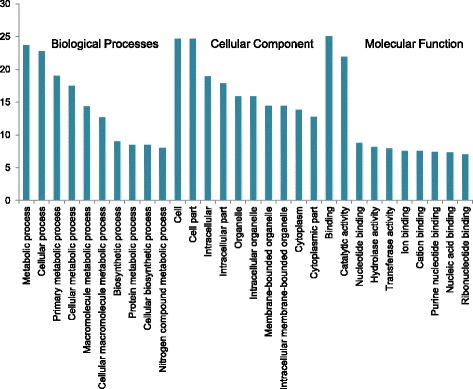
GO classification of *Crocus* unigenes. Bar chart represents percent genes belonging to top 10 biological process, cellular component and molecular function categories

The *Crocus* unigenes were further classified according to gene ontology annotation into three categories viz biological process, cellular component and molecular function (Additional file 4). The top 10 classes from each category are shown in Fig. 1. In the category of biological process most of the genes belonged to metabolic process class (23.7 %) followed by cellular process (22.8 %), primary metabolic process (19 %) and cellular metabolic process (17 %). In case of cellular component, the predominant categories were cell (27 %) and cell part (24.69 %). As far as molecular function is concerned, binding (25 %) and catalytic activity (21.9 %) were the major classes. The GO term abundance results show similarity with previous transcriptome studies, for example, in Gardenia [25], safflower [26] and chrysanthemum [27].

**Fig. 2:**
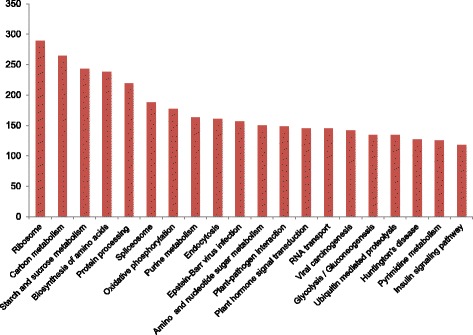
KEGG mapping of *Crocus* unigenes representing number of genes mapped to top 20 classes

In order to elucidate the biochemical pathways represented in *Crocus* transciptome, the sequences were searched against KEGG pathway database. A total of 7319 unigenes (16,958 transcripts) were assigned to 328 KEGG pathways (Additional file 4). The most abundant pathways were ribosome comprising of 289 genes followed by carbon metabolism (264 genes), starch and sucrose metabolism (243 genes) and biosynthesis of amino acids (238 genes). Further, protein processing (219), spliceosome (188) and oxidative phosphorylation (177) also represented significantly higher number of genes. We also identified classes like plant hormone signal transduction (145 genes), plant pathogen interaction (148 genes) and ubiquitin mediated proteolysis (134 genes). The representative top 20 classes are depicted in Fig. 2. In many other transcriptome studies also, the above classes represented the predominant categories. For example, in horse gram the highest number of genes was mapped to ribosome biosynthesis [28] while in chickpea the predominant classes were ribosome, spliceosome and biosynthesis of amino acids [29]. These pathways and the genes involved thereof might be involved in growth and developmental pathways and also in plant response to various environmental cues.

### Identification of genes involved in carotenoid/apocarotenoid biosynthesis

Carotenoid biosynthesis occurs through MEP pathway [30]. The genes encoding enzymes which are involved in this pathway have been isolated in many plant species [31, 32]. In case of *Crocus* also, the pathway has been elucidated to a large extent [4, 12, 33, 34], however, there are still gaps which remain to be filled in terms of the enzymes and the intermediates involved in the pathway. In this study we report identification of many carotenoid/apocarotenoid pathway genes which were known from other plants but not isolated from *Crocus*. This would provide a knowledgebase for understanding the biosynthesis of carotenoids and their subsequent degradation to apocarotenoids in this plant.

**Fig. 3:**
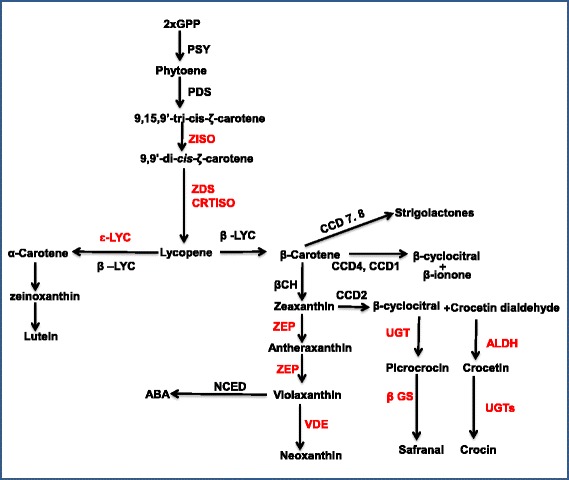
Schematic representation of apocarotenoid pathway showing different enzymes and intermediates involved in the pathway. Genes marked in red are identified first time in this study

Core carotenoid biosynthetic pathway is initiated by condensation of two molecules of geranylgeranyl diphosphate (GGDP) to form phytoene (Fig. 3). This step is catalysed by phytoene synthase (PSY) enzyme. This is the rate limiting step of this pathway [35]. Phytoene, is then desaturated into lycopene by two related enzymes of phytoene desaturase (PDS) and ζ-carotene desaturase (ZDS) in plants [36]. This lycopene is acted upon by two cis-trans isomerases of Z-ISO [37] and CRTISO [38, 39] and is converted from poly-cis-configured phytoene into the all-trans form lycopene. While PSY and PDS were already known from *Crocus* [11], we have identified ZDS, Z-ISO and CRTISO for the first time. Lycopene represents the branching point of this pathway and is cyclized either to form α-carotene by the action of lycopene ε-cyclase (ε-LCY) and lycopene β-cyclase (β-LCY) or is converted to β-carotene by β-LCY alone [40]. β-LCY has been identified from *Crocus* earlier [11, 41], however, ε-LCY from *Crocu*s was not known and has been identified in this study. Futher, α- and β-carotene are hydroxylated to produce lutein and zeaxanthin, respectively by the action of β-ring carotene hydroxylase (BCH). Many isoforms of BCH have been identified from *Crocus* earlier [11]. There is a class of enzymes called carotenoid cleavage dioxygenases (CCDs) which cleave double bonds of carotenoids at different positions resulting in the formation of apocarotenoids. Zeaxanthin acts as substrate for CCD2 which cleaves it into crocetin dialdehyde and β-cyclocitral. CCD2 was very recently identified to be the enzyme which has 7′8 cleavage activity and catalyses the first step in crocin biosynthesis in *Crocus* [33]. Crocetin dialdehyde is converted into crocetin by aldehyde dehydrogenase. In this study we have identified many transcripts encoding aldehyde dehydrogenases which might include the one involved in converting crocetin dialdehyde into crocetin. Further, crocetin and β-cyclocitral are converted into crocin and picrocrocin respectively by UDP-glucosyl transferases. Many transcripts coding for UDP-glucosyl transferases have been identified in this study. So far only a few UDP-glucosyl transferases were known from *Crocu*s [42] and we have added new isoforms to the existing database. The picrocrocin is further converted into saffranal which is supposed to be a beta glucosidase action. Gene encoding this enzyme has not been identified in *Crocus*, so far. Here we have identified many transcripts coding for this enzyme. Therefore, the present study would provide a platform for generating knowledge about the substrate specificities and activities of the enzymes identified so as to understand the apocarotenoid biosynthetic pathway. Further, there are many other CCD isoforms which act on a wide range of substrates, cleave them at different positions and produce a myriad of apocarotenoid products [43]. Analysing the enzyme activities and their substrate specificities would help to identify new compounds. This knowledge can further be useful for designing metabolic engineering strategies for either enhanced production of known compounds or production of new metabolites.

Considering other branch points of this pathway, zeaxanthin is also converted into violaxanthin by zeaxanthin epoxidase (ZEP) which is in turn converted into neoxanthin by neoxanthin synthase (NSY). Violaxanthin and neoxanthin are cleaved by 9-cis-epoxycarotenoid dioxygenase (NCED) to produce xanthoxin, the direct substrate for phytohormone abscisic acid (ABA) synthesis. While NCED from *Crocus* is known [44], the genes encoding the enzymes which act upstream (ZEP and NSY) are identified for the first time in this study. The schematic representation of the carotenoid/apocarotenoid pathway is given in Fig. 3 and the list of pathway genes identified is given in Table 2.

**Table 2:** Genes involved in carotenoid/apocarotenoid pathway

			TMM		RPKM	
Component number	Annotation	Abbreviation	Exp. in stigma	Exp. in flower	Exp. in stigma	Exp. in flower
comp30738_c0_seq8	phytoene synthase	PSY	10.49	1.17	11.9	1.4
comp22417_c0_seq1	phytoene desaturase	PDS	12.48	1.50	26.5	3.3
comp31950_c0_seq2	^a^cis-zeta-carotene isomerase	ZISO	4.17	0.59	4.6	0.7
comp33968_c0_seq1	^a^zeta-carotene desaturase	ZDS	324.18	139.87	88.2	40.1
comp33960_c1_seq3	^a^carotenoid isomerase protein	CRTISO	26.02	2.28	23.0	2.1
comp16731_c1_seq1	lycopene beta-cyclase	β-LYC	1.64	0.46	2.7	0.8
comp17458_c0_seq1	^a^lycopene epsilon-cyclase	ε-LYC	0.89	0.15	2.5	0.5
comp34653_c0_seq16	beta-carotene hydroxylase	BCH	16.81	3.34	9.6	2.0
comp32667_c0_seq2	9-cis-epoxycarotenoid dioxygenase	NCED	1.62	4.35	1.1	3.2
comp21047_c1_seq1	carotenoid cleavage dioxygenase 2	CCD2	2.62	0.15	6.1	0.4
comp8137_c0_seq1	carotenoid cleavage dioxygenase 7	CCD7	0.42	0.00	1.8	0.0
comp8446_c0_seq1	carotenoid cleavage dioxygenase 8b	CCD8b	0.05	0.38	0.1	1.2
comp29644_c0_seq1	carotenoid cleavage dioxygenase 4c	CCD4c	3.83	2.65	5.4	4.0
comp33382_c0_seq3	chromoplast carotenoid cleavage dioxygenase 4b	CCD4b	245.25	27.29	111.6	13.1
comp33944_c0_seq1	^a^Glucosidase	GS	87.77	145.10	80.1	143.3
comp27519_c0_seq1	^a^carotenoid-associated protein	CAP	141.53	59.66	105.3	46.8
comp33950_c0_seq1	Carotenoid 9,10 (9′,10′)-cleavage dioxygenase	CCD1	66.14	19.32	67.4	20.8
comp33382_c0_seq4	Zeaxanthin 7,8 (7′,8′)-cleavage dioxygenase	ZCD	76.17	14.24	29.7	5.9
comp33173_c0_seq2	^a^UDP-glycosyltransferase	UGT	208.34	41.99	8.3	1.5
comp31026_c0_seq1	^a^zeaxanthin epoxidase	ZEP	27.48	12.56	23.8	11.5
comp29444_c0_seq6	^a^violaxanthin de-epoxidase	VDE	8.24	2.79	9.8	3.5

^a^Genes identified first time in the present study

### Identification of transcription factors

**Fig. 4:**
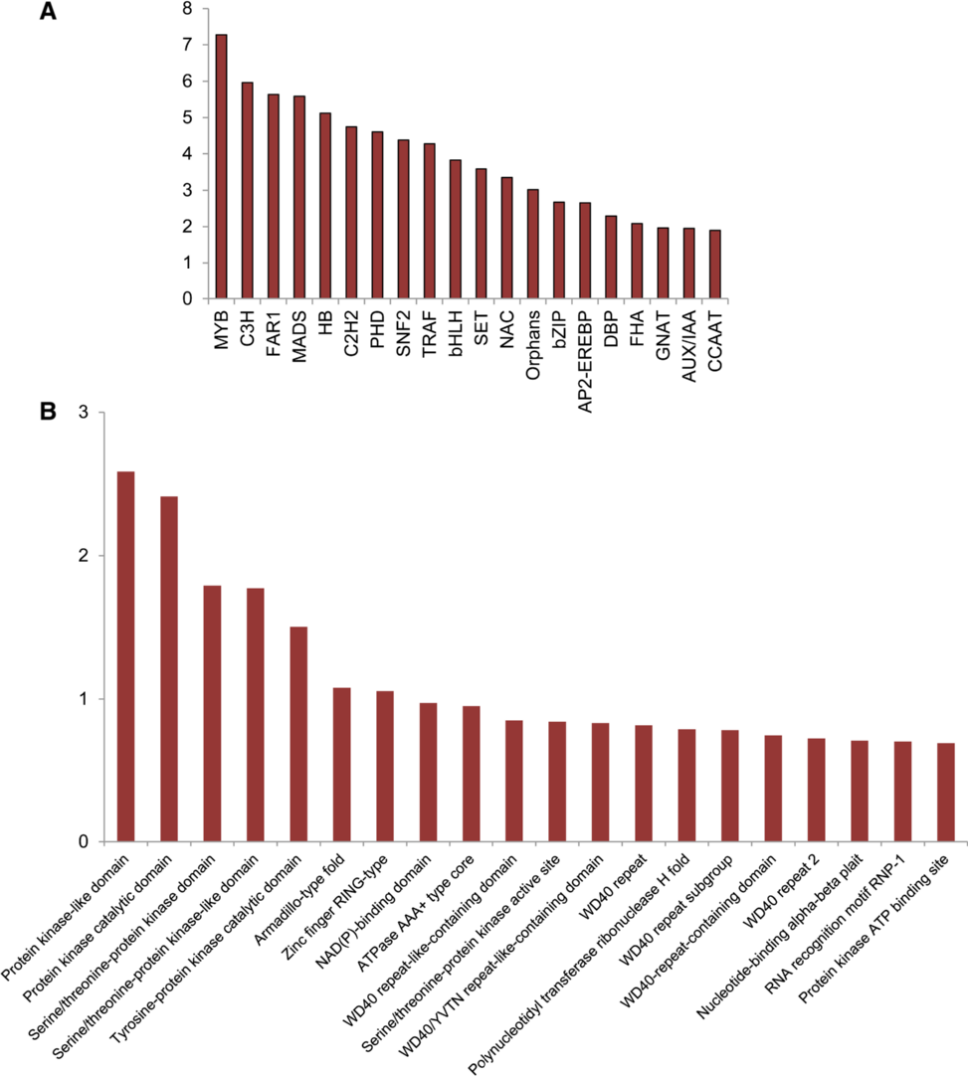
Percent *Crocus* transcripts representing top 20 (**a**) transcription factor families and (**b**) conserved domain families

In order to identify transcription factor encoding genes in *Crocus* transcriptome, homology search was performed against the plant transcription factor database (PlnTFDB). Around 2601 unigenes (5789 transcripts) show similarity to transcription factors representing 76 families (Additional file 5). Out of these, transcription factors belonging to Myb family were most abundant (7.27 %) followed by C3H (5.96 %), FAR1 (5.6 %), MADS box (5.58 %) (Fig. 4a). In many other studies also, the above mentioned classes represented highly expressed transcription factor families. For example, in *Catharanthus roseus*, Myb was the most abundant family [45]. In horse gram, most highly represented transcription factors belonged to C3H, bHLH and AP2 domain families [28]. Transcription factors perform key roles in plant growth and development. They are also involved in regulation of secondary metabolism and also coordinate plant’s response to various environmental cues. Several members of Myb family have been shown to regulate different secondary metabolic pathways [46–48]. Zinc finger proteins of different classes are also involved in regulation of plant secondary metabolism [49]. MADS box genes regulate a range of plant processes including flower development [50] plant reproduction [51] etc. So far regulatory pathway controlling *Crocus* apocarotenoid metabolism is not known. Although many transcription factors were identified from *Crocus* [52, 53], their role in apocarotenoid regulation was not experimentally validated. Recently, a SAND domain ultrapetala transcription factor was isolated from our laboratory and was shown to regulate apocarotenoid biosynthesis in *Crocus* [54]. No other information is available as far regulation of apocarotenoid biosynthesis is concerned. This transcription factor database would therefore, be an important asset to characterize the regulatory pathways of *Crocus* secondary metabolism. This may also provide base for identification of regulators of *Crocus* flower development, its tripartite stigma and would help in addressing other question related to biology of this plant.

### Domain analysis

We aimed to get insight about the molecular mechanism underlying the phenomenon of colour and flavour biogenesis in *Crocus*. GO classification showed the major classes in molecular function comprised of binding, catalytic activity and nucleotide binding. Further, KEGG mapping indicated that genes involved in protein processing, spliceosome and oxidative phosphorylation were significantly represented. In order to extrapolate this information and build up knowledge about the mechanism of action of the genes involved in such activities we performed conserved domain analysis for *Crocus* transcriptome. The major domain classes are given in Fig. 4b. The results showed that most of the genes were those with protein kinase like domain (2.6 %) followed by protein kinase catalytic domain (2.4 %), serine/threonine protein kinase domain (1.8 %), serine/threonine-protein kinase-like domain (1.77 %) and Tyrosine-protein kinase catalytic domain (1.5 %) (Additional file 5). These domains are involved in phosphorylation of proteins at various amino acid residues. Protein modification by phosphorylation and dephosphorylation is a crucial mechanism that controls activity of proteins [55] and as a result regulates important cellular functions in eukaryotes such as cell division, differentiation, signal transduction etc. Predominant presence of genes with different kinase domains in *Crocus* transcriptome is indicative of the fact that even though transcriptional regulation is the central regulatory mechanism for most of the biological processes, diverse post translational events also play important role. These multiple layers of regulation help plant to synchronize properly with developmental stages and environmental cues.

### Differential gene expression, gene ontology and pathway enrichment

**Fig. 5:**
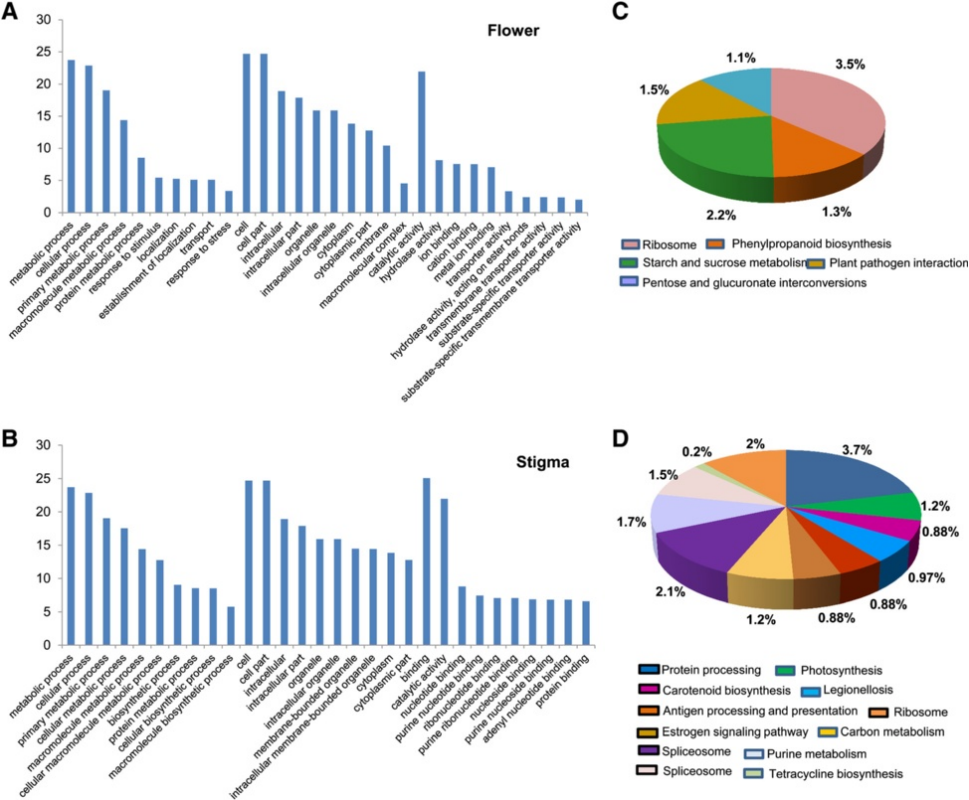
Differential expression analysis of *Crocus* transciptome. **a**, **b** Bar chart showing GO terms associated with genes differentially expressed in flower and stigma. **c**, **d** pie chart representing KEGG classes of flower and stigma upregulated genes

In *Crocus*, the stigma part of the flower has commercial significance because its dried state forms saffron which is the site of biosynthesis and accumulation of apocarotenoids. Although attempts have been made to identify and isolate genes involved in apocarotenoid biosynthesis, little has been done so far. In order to identify genes involved in biosynthesis and regulation of apocarotenoids in *Crocus* stigma, we studied differential gene expression (DGE) in *Crocus* stigma vs. rest of the flower. A total of 3839 transcripts (2741 unigenes) were differentially expressed out of which 2334 transcripts (1746 unigenes) were upregulated in flower and 1505 transcripts (1135 unigenes) in stigma (Additional file 6). In order to identify the major functional categories represented by differentially expressed genes, GO enrichment analysis was carried out (Additional file 7 and Additional file 8). We observed that in flower upregulated genes, the top five classes in biological process were metabolic process, cellular process, primary metabolic process, macromolecule metabolic process and protein metabolic process (Fig. 5a). In case of the genes upregulated in stigma, metabolic process, cellular process, primary metabolic process, cellular metabolic process and macromolecule metabolic process were the top five categories (Fig. 5b). In case of the molecular function category, the top five classes represented by flower upregulated genes were catalytic activity, hydrolase activity, ion binding, cation binding and metal ion binding, while as in case of stigma top five classes were binding, catalytic activity, nucleotide binding, purine nucleotide binding and ribonucleotide binding. Further, genes related to transport were also highly represented in flower while in stigma genes related to nucleotide activity were more prevalent. We also performed KEGG analyses on differentially expressed genes in flower and stigma. The results showed that in case of flower, genes involved in ribosome, starch and sucrose metabolism, plant-pathogen interaction, phenylpropanoid biosynthesis and pentose and glucuronate interconversions were enriched (Fig. 5c) while in case of stigma, genes related to protein processing in endoplasmic reticulum, photosynthesis, carotenoid biosynthesis, legionellosis represented the major classes (Fig. 5d) (Additional file 7 and Additional file 8). Enrichment of carotenoid biosynthesis genes in stigma confirms the fact that biosynthesis of carotenoids and their subsequent degradation into apocarotenoids occurs mainly in stigma.

**Fig. 6:**
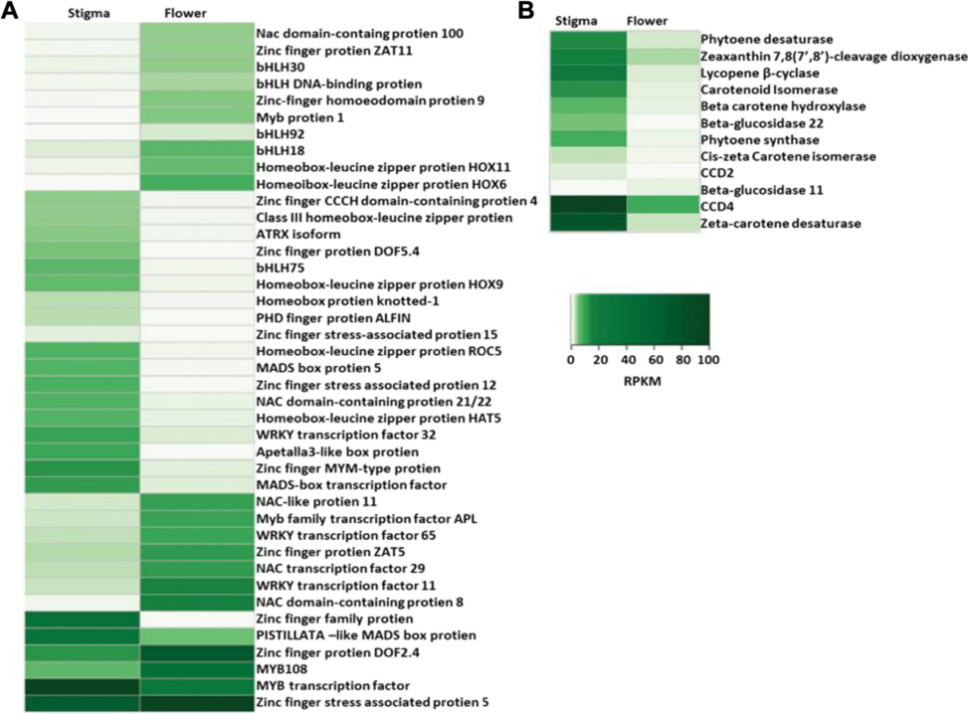
Heat-map showing expression patterns of (**a**) regulatory (**b**) carotenoid pathway genes differentially upregulated in stigma and flower

We also investigated the expression of genes involved in carotenoid/apocarotenoid biosynthesis and observed that most of them were more significantly enriched in stigma (Fig. 6a) which is in accordance with the fact that stigma is the actual site of biosynthesis of apocarotenoids in *Crocus*. For example, *PSY* and *PDS* which catalyse initial steps in carotenoid biosynthetic pathway are expressed more in stigma. Further, *ZISO* and *ZCD* which are involved in converting phytoene into lycopene are also upregulated in stigma. It was quite interesting to see that lycopene *β-LCY* and *BCH* were upregulated in stigma therefore increasing the metabolic flux towards production of zeaxanthin. Earlier reports have also shown increased expression of these genes in *Crocus* stigma [11]. Recently it was shown that *CCD2* cleaves zeaxanthin at 7′8 double bond and results in the formation of crocetin and picrocrocin which are apocarotenoids responsible for color and flavor of saffron [33]. DGE analysis showed that *CCD2* is also upregulated in stigma thereby confirming the earlier reports. Further, another isoform *CCD4b* is also expressed more in stigma. This enzyme cleaves carotenoids like beta-carotene at 9′10 double bond and forms apocarotenoids like β-cyclocitral and β-ionone. Earlier reports have also shown enhanced expression of *CCD4b* in stigma [3]. Thus our results are in agreement with earlier reports and also provide molecular proof for stigma being the actual site of biosynthesis of apocarotenoids which includes crocin, picrorocin, β-cyclocitral and β-ionone.

One of the aims of the present work was to identify transcription factors which regulate the biosynthesis and accumulation of apocarotenoids in *Crocus* in tissue and developmental stage specific manner. Our data suggested that transcription factors belonging to various families like MADS Box, MYB, Zinc finger, WRKY, PHD etc. were differentially expressed in both flower and stigma (Fig. 6b). We observed that different transcription factor families were enriched in stigma and flower. In this context we observed that transcription factors belonging to MADS box were represented more in stigma while NAC, bHLH and WRKY families were enriched more in flower suggesting that they perform specialized roles in different tissues. There were many other transcription factors exhibiting expression in both the tissue types. For example, zinc finger proteins with AN1 domain were more prevalent in stigma upregulated genes while those with ZAT domains were upregulated in flower. This indicates that different members of the same family might play different roles in different tissues or under different conditions.

### Experimental validation of differential expressed genes by quantitative realtime PCR

**Fig. 7:**
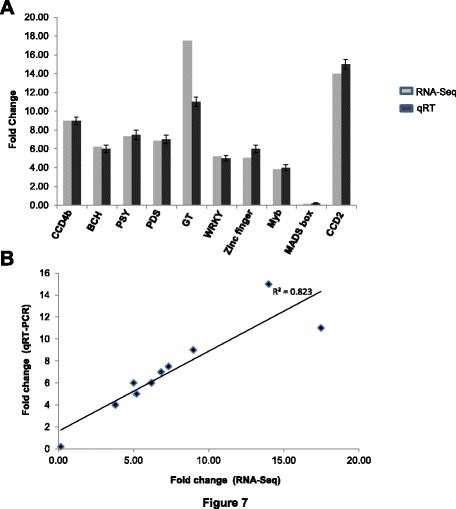
qRT-PCR validation of differentially expressed genes. **a** Expression of 10 genes was validated using qRT-PCR and compared with their expression obtained from RNA-seq. qRT-PCR was performed and values were normalized relative to the expression levels of 18S rRNA in the same cDNA sample. Data are the means (± SE) of three replicates Expression data are presented as expression values of genes in stigma sample relative to their expression in flower sample (**b**) correlation of gene expression results obtained from qPCR and RNA-seq

qRT-PCR of ten selected genes was performed in order to validate the differential gene expression obtained by RNA-seq. The genes selected were CCD4b, CCD2, BCH, PSY, PDS, GT, Zinc-finger, MADS box, Myb and, WRKY. The results indicated that expression pattern as obtained by qRT-PCR corroborated with that obtained by RNA-seq for all the genes (Fig. 7a). This confirmed the reliability of RNA-seq data. We also performed statistical analysis on the data obtained for these genes from RNA-seq and qPCR and observed that there was very good correlation between the two (correlation coefficient 0.8) (Fig. 7b).

### Phylogenetic analyses of *CCD* gene family

**Fig. 8:**
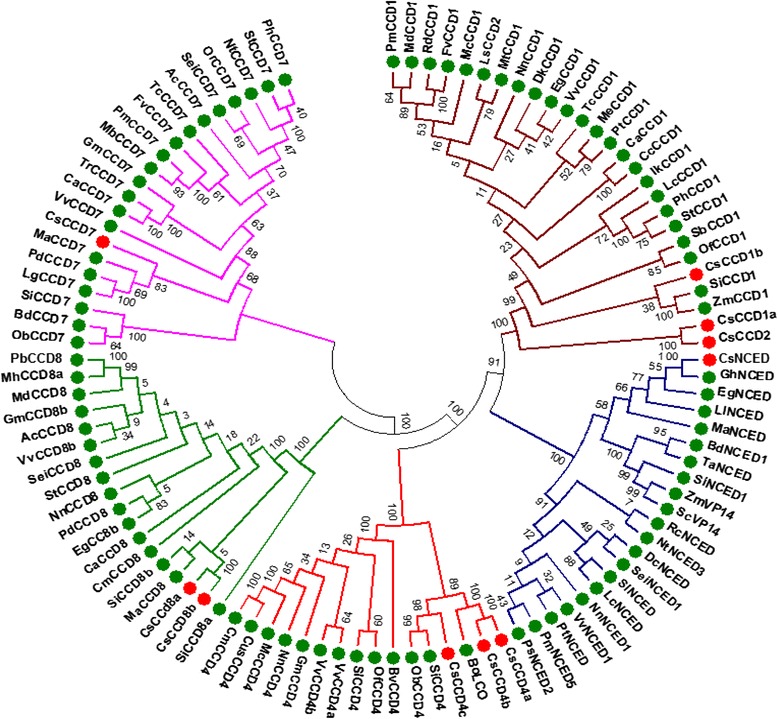
Phylogenetic tree for CCD proteins. Tree was constructed by aligning 103 CCD proteins from 53 plant species including *Crocus* using the MUSCLE program and subjected to phylogenetic ana lysis by the distance with neighbor-joining method using the MEGA6.05 software. The numbers on the nodes indicate the bootstrap values after 1000 replicates

Carotenoid cleavage dioxygenases (CCDs) form a group of enzymes which are involved in cleavage of carotenoids leading to the production of apocarotenoids. CCDs are specific to the double bond they cleave but often exhibit substrate promiscuity which is responsible for the diversity of apocarotenoids found in nature. Apocarotenoids play role in many aspects of plant growth and development. The CCD family is ancient and has its members present in bacteria, plants and animals. Members of the CCD family share several characteristics like, they require a Fe^2+^ for catalytic activity; they have four conserved histidines which coordinate iron binding and they contain a conserved peptide sequence at their carboxyl terminus [56]. In order to get an insight about the phylogenetic relationship between various CCDs, a neighbour-joining phylogenetic tree was constructed with 103 CCD genes from 53 plant species. The amino acid sequences from various CCDs were grouped into five clusters, named CCD1, CCD4, CCD7, CCD8 and NCED (Fig. 8). In each of these clusters, CCDs were present in two groups corresponding to monocotyledon and dicotyledon species. Further, genes or isoforms within the CCD sub-family and belonging to same species were grouped in the same branch, for example, *Crocus sativus* CCD4a/b, CCD8a/b. Phylogenetic analysis also revealed that two major duplications had occurred in CCD subfamilies. CCD duplication, which occurred in the moss lineages, ultimately led to the emergence of two lineages that evolved into CCD7 and CCD8. This result suggests that, CCD7/8 genes had similar evolutionary trends than CCD1 and CCD4 sub-family. In general, CCDs belonging to a particular cluster have similar cleavage activity. Recently a new CCD isoform (CsCCD2) was identified from *Crocus sativus*, which plays an important role in crocin biosynthesis. CsCCD2 clusters with CCD1 subfamily but is distinct from CCD1 subfamily as far as its catalytic activity is concerned [33]. This suggests that CCD2 might have evolved from CCD1 and developed different cleavage site specificity. Thus phylogenetic analysis may help in understandin g functional diversity of CCD gene family.

## Conclusions

In the present study, *Crocus sativus* transcriptome was sequenced, assembled and annotated. The database led to the identification of many new candidate genes involved in carotenoid/apocarotenoid pathway. Identification of transcription factors provides a platform for unravelling the regulatory pathway of *Crocus* flower development and apocarotenoid biosynthesis. Differential gene expression and pathway mapping confirmed enrichment of apocarotenoid pathway genes in stigma thus confirming that stigma is the site of apocarotenoid biosynthesis. The transcript resource generated would therefore facilitate and enhance our understanding of biosynthetic pathway of carotenoids and their subsequent tailoring into apocarotenoids and the mechanism that regulates carotenoid-apocarotenoid metabolic flux.

### Availability of supporting data

The Illumina sequence data have been submitted as Bioproject [PRJNA277895] to NCBI sequence read archive under accession number [SRP056059]. All the other supporting data are included as additional files.

## Additional files

Additional file 1:
**Sequence of the primers used in this study.** (DOCX 15 kb) Additional file 2:
**Accession numbers of the genes used for phylogenetic analysis.** (DOC 102 kb) Additional file 3:
**Functional annotation of** ***Crocus*** **transcriptome.** (XLSX 6986 kb) Additional file 4:
**GO and KEGG classes of** ***Crocus*** **transcripts.** (XLSX 18460 kb) Additional file 5:
**Details of transcription factor and conserved domain families.** (XLSX 2674 kb) Additional file 6:
**Details of differentially expressed transcripts and flower and stigma upregulated genes.** (XLSX 1168 kb) Additional file 7:
**GO and KEGG classes in flower upregulated genes.** (XLSX 23 kb) Additional file 8:
**GO and KEGG classes in stigma upregulated genes.** (XLSX 25 kb)

## Abbreviations

GO: Gene ontology
KEGG: Kyoto enclyclopedia of genes and genomes
CCD: Carotenoid cleavage dioxygenase
GGDP: Geranylgeranyl diphosphate
PSY: Phytoene synthase
PDS: Phytoene desaturase
