# Supplementary material for: Comprehensive transcriptome analysis of Crocus sativus for discovery and expression of genes involved in apocarotenoid biosynthesis
Source: BMC Genomics. 2015 Sep 15;16(1):698. doi: 10.1186/s12864-015-1894-5 (PMC4570256; doi:10.1186/s12864-015-1894-5)
Supplement: Additional file 1: — Sequence of the primers used in this study. (DOCX 15 kb) [file 12864_2015_1894_MOESM1_ESM.docx]

**Additional file 1 List of primer sequences**

| S.No. | Name of Primer | Sequence of Primer |
| --- | --- | --- |
| 1 | CCD4b-F | ATCTCATCACCCGACACTCC |
| 2 | CCD4b-R | TTG GGT TGG GAC CGT TGC GG |
| 3 | CCD2-F | CCD2-F GTCAGTTGAGTTCTGCAGAGGT |
| 4 | CCD2-R | CCD2-R TAGAGTAAAGTGGAATTCAAT |
| 5 | BCH-F | ATGGGCCCCACAGGGAACCG |
| 6 | BCH-R | GGA GGG CCC TTT CGA GCT CAA |
| 7 | PSY-F | TTC CCG TCA TGG GAA TTG CAC C |
| 8 | PSY-R | AAG GCC TGA TTG TGC AAG CTC |
| 9 | PDS-F | TAG CTG AGT CAT GGA ATA CTC |
| 10 | PDS-R | TTG CGC GGA GTG AGA AGT GC |
| 11 | GT-F | TCACGTCGGGTTTGTCTCGAA |
| 12 | GT-R | CAATAGCACTCCCACATGC |
| 13 | WRKY-F | GCCAGCCAGTACTGCTCTCCTT |
| 14 | WRKY-R | AGATTCCAGTACTTTCTCACCAC |
| 15 | ZnFn-F | AACTGCAATCAGCTCGACTTCC |
| 16 | ZnFn-R | TTGCATGCCGTTCGAGGACTGCC |
| 17 | Myb-F | TACCTGAGGCCGGACGTTCGGA |
| 18 | Myb-R | CATCGATGCCATTATTGGAGT |
| 19 | MADS box-F | ATT CCA TCT ACT CCG TTC TCC C |
| 20 | MADS box-R | AAC CCA TCA ATC CAA TGG CTG C |
| 21 | 18S-F | ACGAAACCCCGGCGCAGTGGGC |
| 22 | 8S-R | TCGCTACGTTCTTCATCGATGCG |
