# Supplementary material for: Comprehensive transcriptome analysis of Crocus sativus for discovery and expression of genes involved in apocarotenoid biosynthesis
Source: BMC Genomics. 2015 Sep 15;16(1):698. doi: 10.1186/s12864-015-1894-5 (PMC4570256; doi:10.1186/s12864-015-1894-5)
Supplement: Additional file 2: — Accession numbers of the genes used for phylogenetic analysis. (DOC 102 kb) [file 12864_2015_1894_MOESM2_ESM.doc]

**Additional file 2** Accession numbers of the CCD genes used in the phylogenetic analysis

| **S. No.** | **Organism** | **CCD Group** | **NCBI accession no.** |
| --- | --- | --- | --- |
| 1 | *Coffea arabica* | *Ca*CCD1 | gi|76560804 |
| 2 | *Coffea canephora* | *Cc*CCD1 | gi|76560796 |
| 3 | *Crocus sativus* | *Cs*CCD1a | gi|17907098 |
| 4 | *Crocus sativus* | *Cs*CCD1b | gi|667794902 |
| 5 | *Diospyros kaki* | *Dk*CCD1 | gi|379063012 |
| 6 | *Eucalyptus grandis* | *Eg*CCD1 | gi|702426515 |
| 7 | *Fragaria vesca* | *Fv*CCD1 | gi|470141642 |
| 8 | *Ipomoea sp. Kenyan* | *Ik*CCD1 | gi|262036882 |
| 9 | *Lactuca sativa* | *Ls*CCD1 | gi|84579416 |
| 10 | *Lycium chinense* | *Lc*CCD1 | gi|569533233 |
| 11 | *Malus domestica* | *Md*CCD1 | gi|657966949 |
| 12 | *Manihot esculenta* | *Me*CCD1 | gi|307592509 |
| 13 | *Medicago truncatula* | *Mt*CCD1 | gi|195972576 |
| 14 | *Momordica charantia* | *Mc*CCD1 | gi|408794951 |
| 15 | *Nelumbo nucifera* | *Nc*CCD1 | gi|720078124 |
| 16 | *Osmanthus fragrans* | *Os*CCD1 | gi|294679657 |
| 17 | *Petunia x hybrida* | *Ph*CCD1 | gi|49659730 |
| 18 | *Populus trichocarpa* | *Pt*CCD1 | gi|566186637 |
| 19 | *Prunus mume* | *Pm*CCD1 | gi|645250526 |
| 20 | *Rosa x damascena* | *Rd*CCD1 | gi|163881523 |
| 21 | *Scutellaria baicalensis* | *Sb*CCD1 | gi|508083244 |
| 22 | *Setaria italica* | *Si*CCD1 | gi|514755705 |
| 23 | *Solanum tuberosum* | *St*CCD1 | gi|565357220 |
| 24 | *Theobroma cacao* | *Tc*CCD1 | gi|590724612 |
| 25 | *Vitis vinifera* | *Vv*CCD1 | gi|387763758 |
| 26 | *Zea mays* | *Zm*CCD1 | gi|107857142 |
| 41 | *Beta vulgaris* | *Bv*CCD4 | gi|731332791 |
| 27 | *Bixa orellana* | *Bo*LCCO | gi|40642651 |
| 28 | *Crocus sativus* | *Cs*CCD4a | gi|188529515 |
| 29 | *Crocus sativus* | *Cs*CCD4b | gi|188529517 |
| 30 | *Crocus sativus* | *Cs*CCD4c | gi|346722086 |
| 31 | *Cucumis melo* | *Cm*CCD4 | gi|659101954 |
| 32 | *Cucumis sativus* | *Cus*CCD4 | gi|449502172 |
| 33 | *Glycine max* | *Gm*CCD4 | gi|356495283 |
| 34 | *Momordica charantia* | *Mc*CCD4 | gi|40879495 |
| 35 | *Nelumbo nucifera* | *Nn*CCD4 | gi|720061328 |
| 36 | *Oryza brachyantha* | *Ob*CCD4 | gi|573920306 |
| 37 | *Osmanthus fragrans* | *Os*CCD4 | gi|164551336 |
| 38 | *Setaria italica* | *Si*CCD4 | gi|514716865 |
| 39 | *Solanum lycopersicum* | *S*lCCD4 | gi|723726437 |
| 40 | *Vitis vinifera* | *Vv*CCD4a | gi|387763754 |
| 41 | *Vitis vinifera* | *Vv*CCD4b | gi|532020843 |
| 42 | *Actinidia chinensis* | *Ac*CCD7 | gi|310896479 |
| 43 | *Brachypodium distachyon* | *Bd*CCD7 | gi|357168137 |
| 44 | *Cicer arietinum* | *Ca*CCD7 | gi|502166602 |
| 45 | *Crocus sativus* | *Cs*CCD7 | gi|663160914 |
| 46 | *Elaeis guineensis* | *Eg*CCD7 | gi|743829381 |
| 47 | *Fragaria vesca* | *Fv*CCD7 | gi|470142568 |
| 48 | *Glycine max* | *Gm*CCD7 | gi|356495887 |
| 49 | *Malus baccata* | *Mb*CCD7 | gi|586616522 |
| 50 | *Musa acuminata* | *Ma*CCD7 | gi|695074514 |
| 51 | *Nicotiana tomentosiformis* | *Nt*CCD7 | gi|697167571 |
| 52 | *Orobanche ramosa* | *Or*CCD7 | gi|350605181 |
| 53 | *Oryza brachyantha* | *Ob*CCD7 | gi|573940410 |
| 54 | *Petunia x hybrida* | *Ph*CCD7 | gi|261863854 |
| 55 | *Phoenix dactylifera* | *Pd*CCD7 | gi|672144503 |
| 56 | *Prunus mume* | *Pm*CCD7 | gi|645253531 |
| 57 | *Sesamum indicum* | *Sei*CCD7 | gi|747045524 |
| 58 | *Setaria italica* | *Si*CCD7 | gi|514803094 |
| 59 | *Solanum tuberosum* | *St*CCD7 | gi|565362379 |
| 60 | *Theobroma cacao* | *Tc*CCD7 | gi|590723573 |
| 61 | *Trifolium repens* | *Tr*CCD7 | gi|601036844 |
| 62 | *Vitis vinifera* | *Vv*CCD7 | gi|225453464 |
| 63 | *Actinidia chinensis* | *Ac*CCD8 | gi|310896477 |
| 64 | *Cicer arietinum* | *Ca*CCD8 | gi|502131934 |
| 65 | *Crocus sativus* | *Cs*CCD8a | gi|663160938 |
| 66 | *Crocus sativus* | *Cs*CCD8b | gi|663160921 |
| 67 | *Cucumis melo* | *Cm*CCD8 | gi|659088500 |
| 68 | *Elaeis guineensis* | *Eg*CCD8 | gi|743796224 |
| 69 | *Glycine max* | *Gm*CCD8b | gi|571449734 |
| 70 | *Malus domestica* | *Md*CCD8 | gi|658050868 |
| 71 | *Malus hupehensis* | *Mh*CCD8 | gi|683524549 |
| 72 | *Musa acuminata* | *Ma*CCD8 | gi|695038997 |
| 73 | *Nelumbo nucifera* | *Nn*CCD8 | gi|719979724 |
| 74 | *Phoenix dactylifera* | *Pd*CCD8 | gi|672169213 |
| 75 | *Pyrus x bretschneideri* | *Pb*CCD8 | gi|694382949 |
| 76 | *Sesamum indicum* | *Sei*CCD8 | gi|747041043 |
| 77 | *Setaria italica* | *Si*CCD8a | gi|514790829 |
| 78 | *Setaria italica* | *Si*CCD8 | gi|514790825 |
| 79 | *Solanum tuberosum* | *St*CCD8 | gi|565387981 |
| 80 | *Vitis vinifera* | *Vv*CCD8 | gi|225429936 |
| 81 | *Brachypodium distachyon* | *Bd*NCED1 | gi|357120366 |
| 82 | *Crocus sativus* | *Cs*NCED | gi|188011139 |
| 83 | *Daucus carota* | *Dc*NCED | gi|79155257 |
| 84 | *Elaeis guineensis* | *Eg*NCED | gi|743761243 |
| 85 | *Gladiolus hybrid* | *Gh*NCED | gi|334361424 |
| 86 | *Lilium longiflorum* | *ll*NCED | gi|478348137 |
| 87 | *Lilium speciosum* | *Ls*NCED | gi|260176996 |
| 88 | *Lycium chinense* | *Lc*NCED | gi|727924617 |
| 89 | *Musa acuminata* | *Ma*NCED | gi|695038388 |
| 90 | *Narcissus tazetta* | *Nt*NCED | gi|478427863 |
| 91 | *Nelumbo nucifera* | *Nn*NCED1 | gi|720098791 |
| 92 | *Nicotiana tabacum* | *Nt*NCED3 | gi|399932285 |
| 93 | *Pisum sativum* | *Ps*NCED2 | gi|22335699 |
| 94 | *Populus trichocarpa* | *Pt*NCED | gi|224114842 |
| 95 | *Prunus mume* | *Pm*NCED | gi|645238050 |
| 96 | *Ricinus communis* | *Rc*NCED | gi|255557269 |
| 97 | *Sesamum indicum* | *Sei*NCED | gi|747100101 |
| 98 | *Setaria italica* | *Si*NCED1 | gi|514823384 |
| 99 | *Solanum lycopersicum* | *Sl*NCED | gi|350535531 |
| 100 | *Sorghum bicolor* | *Sc*VP14 | gi|559100101 |
| 101 | *Triticum aestivum* | *Ta*NCED | gi|431812563 |
| 102 | *Vitis vinifera* | *Vv*NCED | gi|526118124 |
| 103 | *Zea mays* | *Zm*VP14 | gi|414871750 |
